# Emerging roles of ADAM6 and PRSS1 as novel diagnostic/prognostic biomarkers for acute lymphoblastic and myeloid leukemia in adults

**DOI:** 10.1186/s12885-025-14292-9

**Published:** 2025-05-17

**Authors:** Heba M. Anis, Nahed M. Rakha, Dina H. Kassem, Amany M. Kamal

**Affiliations:** 1 Children’s Cancer Hospital 57357, Cairo, Egypt; 2 Clinical Hematology Unit, Internal Medicine Department, Faculty of Medicine, Ain Shams University, Cairo, Egypt; 3 Biochemistry Department, Faculty of Pharmacy, Ain Shams University, Cairo, Egypt

**Keywords:** Acute lymphoblastic leukemia, Acute myeloid leukemia, ADAM6, Cancer biomarkers, PRSS1, Tumor microenvironment

## Abstract

**Background:**

Acute leukemia is an aggressive, highly heterogeneous hematological malignancy. A Disintegrin And Metalloproteinase Domain-6 (ADAM6), a member of ADAMs family, has emerged recently as a potential novel player in pediatric acute lymphoblastic leukemia (ALL), and its function remains largely elusive. Serine Protease-1 (PRSS1) is another emerging molecular mediator in cancer development. However, its role in acute leukemia has not been adequately studied. Interestingly, ADAM6 and PRSS1 were identified among the genes with the highest percentage of chromosomal changes in profiled B-cell precursor ALL patients. Both are emerging novel mediators of extracellular matrix (ECM) remodeling. Thus, this study was designed to investigate the roles of ADAM6 and PRSS1 in ALL and acute myeloid leukemia (AML) in adults.

**Methods:**

Adult patients with de novo ALL (*n* = 36), de novo AML (*n* = 40), and healthy control subjects (*n* = 55) were enrolled in this study. Circulating serum levels of ADAM6 and PRSS1 were measured by ELISA technique.

**Results:**

Serum levels of ADAM6 were significantly higher in ALL and AML patients compared to healthy control subjects (208.7(178.3–337.3), 186.4(155.3–479.6), and 78.6(55.8–101.8) pg/ml, *p* < *0.0001*), respectively. Whereas, serum levels of PRSS1 were found to be significantly lower in ALL and AML patients compared to healthy controls (175.1(153.7–232.2), 177.9(145.3–206.4), and 247.5(204.3–375.3) ng/ml, *p* < *0.0001*), respectively. Both ADAM6 and PRSS1 exhibited a very good diagnostic potential by ROC analyses. ADAM6 levels significantly varied between CD22^+^/CD22^−^ and CD45^+^/CD45^−^, while PRSS1 levels significantly varied between HLA-DR^+^/HLA-DR^−^ ALL patients, suggesting their prognostic implications. Also, ADAM6 and PRSS1 were found to be significantly correlated with each other.

**Conclusion:**

The results of the current study portray ADAM6 and PRSS1 as new potential diagnostic/prognostic biomarkers and potential therapeutic targets in adult acute leukemia patients, and shed light on their role as novel interrelated mediators possibly implicated in tumor micro-environment remodeling.

**Graphical Abstract:**

This figure was created in BioRender. Kassem, D. (2025) https://BioRender.com/tf8iofn.

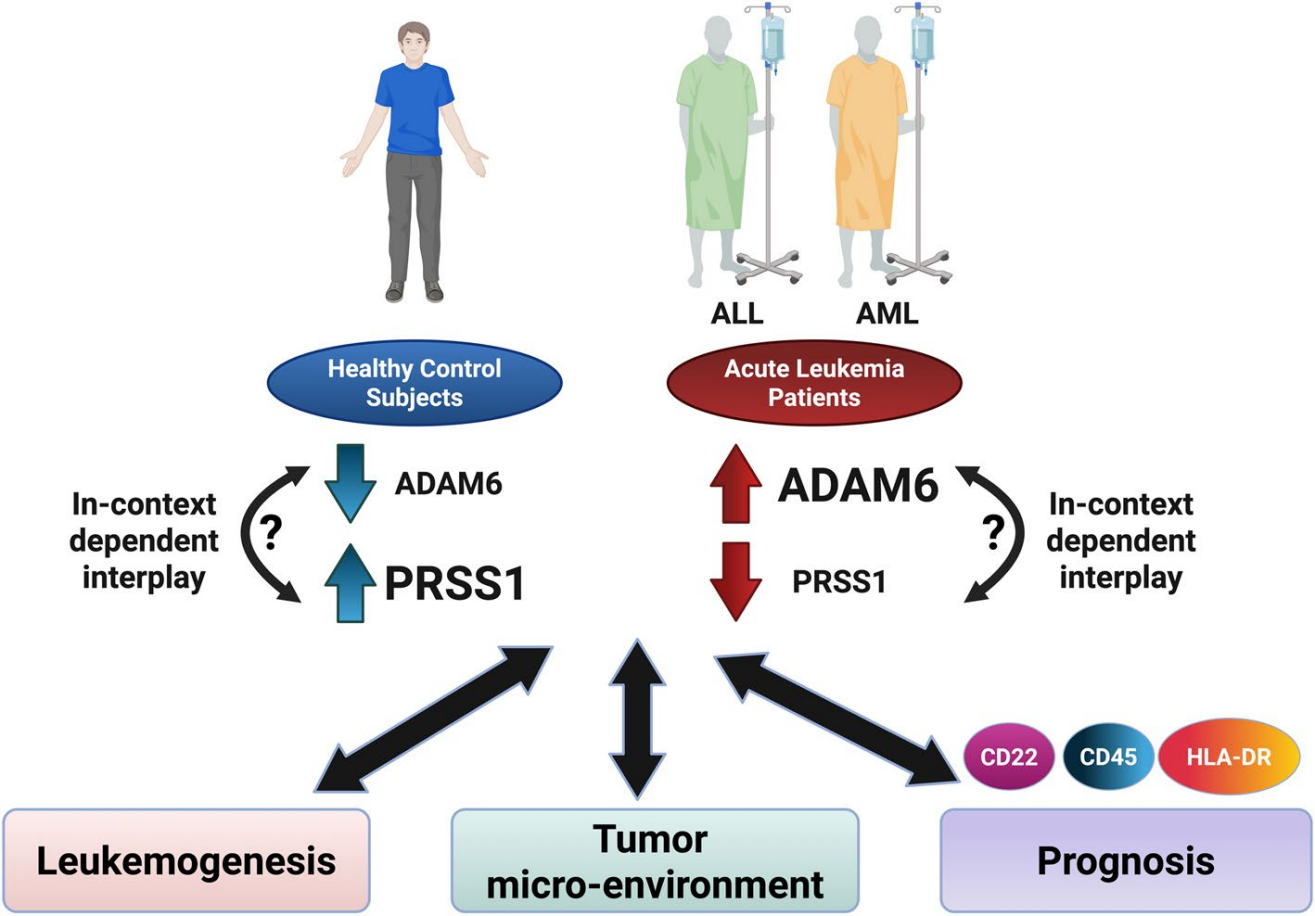

**Supplementary Information:**

The online version contains supplementary material available at 10.1186/s12885-025-14292-9.

## Introduction

Leukemia is defined as a heterogeneous group of haemopoietic cancers that consists of many diverse and biologically distinct subtypes [1]. It is ranked as the thirteenth common cancer type according to global incidence, and the tenth based on mortality worldwide [2]. Acute leukemia generally appears suddenly and progresses rapidly over a very short time. It is characterized by abnormal differentiation and proliferation of hematopoietic stem cells (HSCs), resulting in immature precursors accumulation in the bone marrow and peripheral blood [3, 4]. Both acute myeloid leukemia (AML) and acute lymphoblastic leukemia (ALL) pose major challenges in the field of hematological malignancies [5].

AML is a heterogeneous myeloid neoplasm characterized by the clonal expansion of undifferentiated myeloid progenitor cells (myeloblasts), causing their accumulation in the bone marrow and peripheral blood, together with impaired hematopoiesis [6]. It is one of the most common types of leukemia in adults; almost three to four times more common in adults than ALL [6, 7]. Regretfully, despite advancements in therapeutic approaches over the past decades, prognosis remains relatively suboptimal, especially among older populations with a mere 15% five-year survival rate [5]. As for ALL, it is an aggressive hematological malignancy characterized by uncontrolled proliferation of immature abnormal B and T-lymphocytes and their progenitors (lymphoblasts), ultimately leading to bone marrow failure [5]. It is the most common type of leukemia in the pediatric population, accounting for about 80% of cases in children [7, 8], but most deaths from ALL occur in adults [9]. Although treatment of ALL is relatively considered a success story in pediatric oncology, the cure rate in adults lags far behind that in children [10, 11].

The appropriate diagnostic work-up of acute leukemia patients helps to assess the initial extent of disease and stability of the patient, and provides the required information for proper risk stratification [5, 8]. Such information includes patient age, white blood cell count at diagnosis, leukemia immunophenotype [12] presence or absence of extramedullary disease, and blast cytogenetic abnormalities [13]. There is a crucial need for novel diagnostic/prognostic biomarkers and therapeutic targets for AML and ALL to improve early disease detection and to improve overall patients’ survival [8].

Members of a disintegrin and metalloproteinases (ADAMs) family of proteins have emerged as key players in cancer development and progression [14]. Typically, ADAMs are formed of a pro-domain, a metalloproteinase, disintegrin, cysteine-rich, and transmembrane domains [15]. They can either be fixed on the cell membrane by their transmembrane domain or secreted within the circulation [15, 16]. Nearly 40–50% of ADAMs act as functional proteases, as indicated by the presence of the characteristic HEXGHXXGXXHD motif within their catalytic domain [17]. Shedding of membrane proteins is one of the major functions of ADAMs; their substrates include adhesion molecules, receptors, as well as growth factors, chemokines, and extracellular matrix components [18, 19]. On the other hand, several members of the ADAMs family have been regarded as non-proteolytic mediators and considered as probable pseudogenes, including ADAM6, located in chromosome 14 (14q32.33) whose function in normal physiology or cancer has not been adequately studied [19, 20]. It’s noteworthy here that several members of the ADAM family were previously reported to be interrelated with acute leukemia including ADAM3 A [21], ADAM10 [22], ADAMTS2 [23], and ADAM28 [24, 25]. For example, the ectodomain sheddase ADAM10 was previously reported to play a role in regulating Notch signaling, thereby being implicated in leukemia pathogenesis, and various strategies to inhibit/downregulate ADAM10 were suggested to possibly counteract aberrant Notch signaling in T-ALL and decrease cancer progression [26, 27].

Importantly, deletions of ADAM6 have been reported to have prognostic implications in pediatric ALL, specifically the B-cell precursor (BCP) ALL subtype [20] and chronic lymphoblastic leukemia [28]. Moreover, ADAM6 gene homozygous deletions were found to be significantly associated with unique microRNA expression patterns upon in silico analysis of publicly available ALL datasets [20]. However, the potential role of ADAM6 has not been investigated in AML or ALL in adults.

Another potential biomarker in cancer that has attracted much interest lately is serine protease 1 (PRSS1, *PR*otea*S*e *S*erine *1*), which codes for human cationic trypsinogen [29]. Under physiological conditions, the pro-enzyme trypsinogen is specifically and highly expressed in the pancreas, and afterwards converted into its active form trypsin by cleavage of trypsinogen activation peptide, usually in the small intestine by the action of enterokinase or auto-catalytically by trypsin itself [30]. Variants and/or mutations in the *PRSS1* gene have been found to be associated with pancreatitis [31–33] and pancreatic adenocarcinoma [34, 35]. Two distinct pathological pathways for chronic pancreatitis have been identified based on *PRSS1* variants; the trypsin-dependent pathway in which several *PRSS1* variants increase trypsinogen (auto)activation and/or trypsin stability, and the misfolding-dependent pathway in which *PRSS1* missense variants induce the formation of misfolded proteins that in turn induce endoplasmic reticulum (ER) stress pathway [36]. Importantly, PRSS1 variants (rs10273639) have been reported to be associated with the risk of asparaginase-associated pancreatitis in children with ALL [37], and deletions from the 7q34 chromosomal region of the *PRSS1* gene detected by array analysis have been reported to potentially play a role in ALL pathogenesis [38].

It’s important to point here that an interesting report by Alsuwaidi and coworkers identified both *ADAM6* and *PRSS1* among the genes with the highest percentage of chromosomal changes in profiled B-cell precursor ALL patients, and identified PRSS1 among the potential novel players mediating *ADAM6* gene homodeletion effects in pediatric ALL [20]. Furthermore, both ADAM6 and PRSS1 are emerging novel mediators of extracellular matrix (ECM) and tumor micro-environment (TME) remodeling [39–41]. Thus, in the current study, we sought to investigate the potential role of ADAM6 and PRSS1 in leukemogenesis as well as their potential as diagnostic and/or prognostic biomarkers in adult patients with ALL and AML. As far as our knowledge goes, this is the first study investigating their potential role and interrelation in adult patients with acute leukemia.

## Subjects and methods

### Subjects

The study was conducted from March 2022 to May 2023 at the Clinical Hematology and Stem Cell Transplantation Unit, Ain Shams University Hospitals, Cairo, Egypt. It is noteworthy that before conducting the study, we carried out a priori power analysis using G power software, and got a preliminary indication for the minimum suitable total sample size which would enable us to detect a significant difference (If exists) between the acute leukemia and control groups with a reasonable power (≥ 0.85), and a medium effect size. The total sample size suggested was around 122 subjects. Accordingly, we proceeded with the recruitment of a total of 40 de novo adult AML patients (15 females and 25 males) with median age of 28 years and 36 de novo ALL patients (19 females and 17 males) with median age of 41 years. Patients were generally followed up for 12 months after diagnosis. The study also included 55 age and gender-matched healthy control subjects (14 females and 41 males) with median age of 37 years. Informed consent was obtained from every participant according to the Human Ethical Review committee, Faculty of Pharmacy, Ain Shams University, Cairo, Egypt (Msc: no. 85), and the study was carried out in accordance with the regulations and recommendations of the Declaration of Helsinki. At the end of the study, a post-hoc power analysis was carried out using G power software in the light of the numbers of actually enrolled subjects and reported levels of ADAM6 and/or PRSS1 in various subgroups which revealed an approximate power of (> 0.9).

### Methods

#### Blood sampling

8 mLs of peripheral blood were taken from the patients before starting their treatment protocols. Samples were collected on EDTA vacutainer tubes for complete blood count (CBC) analysis, immunophenotyping, and cytogenetic studies, and on plain gel vacutainer tubes for serum preparation. Briefly, samples were collected over gel vacutainers to ensure efficient and complete clotting. The samples were centrifuged within 1 h after collection from the patients and immediately stored at − 80 °C. Lipemic or hemolyzed samples were discarded and not included in the study. The separated sera were further divided into aliquots and stored at − 80 °C.

#### Complete blood count (CBC) and immunophenotyping analyses

Hemoglobin (Hgb), total leukocyte count (TLC), platelet count (PLT), peripheral blood blast percentage and absolute peripheral blood blast count, absolute neutrophil count, and absolute lymphocyte count were assessed using Z2 TM Coulter Counter®, Analyzer, Coulter Electronics, USA. Immunophenotyping was performed via flow cytometry to detect the expression of various cluster of differentiation (CD) surface markers using diagnostic kits supplied by Beckman Coulter, Fullerton, USA [42].

#### Cytogenetic analysis

Fluorescence in situ hybridization (FISH) technique was used to determine cytogenetic abnormalities by using Locus-specific identifiers (LSI) DNA probe (fluorophore labeled) provided by Abbott Molecular, USA, to detect the cytogenetic karyotype for every patient (45 X, 46 XX, 46 XY, or trisomy). Moreover, fluorescence microscopy was used to perform dual-color FISH and visualize hybridization signals [43, 44].

#### Determination of serum levels of ADAM6 and PRSS1

Serum levels of ADAM6 and PRSS1 were determined by enzyme-linked immunosorbent assay (ELISA) using commercially available kits. Serum PRSS1 levels were determined using Human PRSS1 ELISA kit *(Bioassay Technology Laboratory, catalog No. E3765Hu, China*), with intra-assay variability of < 8% and inter-assay variability of < 10%*.* Serum ADAM6 levels were determined using Human ADAM6 ELISA kit *(Glory Science Co., catalog No.. I4921, China),* with intra-assay variability of < 9% and inter-assay variability of < 15%. The levels of variability for both kits are pretty typical for research-use ELISA kits. All ELISA procedures were done according to the manufacturers’ instructions using chromate micro-plate reader *(Awareness Technology, USA),*

#### Statistical methods

Statistical analysis was done using IBM© SPSS© Statistics version 24 (IBM© Corp. Armonk, NY) and MedCalc© version 20.218 (MedCalc Software Ltd, Ostend, Belgium; https://www.medcalc.org; 2023). Graphs were plotted using GraphPad Prism (version 10.3.1). Normality of numerical data distribution was examined using the D’Agostino-Pearson test. Normally distributed numerical data are presented as mean ± SD, and intergroup differences are compared using One-way ANOVA with application of the Tukey test for post hoc comparisons if needed. Non-normally distributed numerical data are presented as median (interquartile range), and intergroup differences are compared using the Mann–Whitney or Kruskal–Wallis followed by Dunn’s test, according to the number of groups. The Jonckheere-Terpstra test was used to compare non-normally distributed numerical data across ranked categories. The Conover test was applied for post hoc comparisons after the Kruskal–Wallis test or the Jonckheere-Terpstra test if needed. Categorical variables are presented as ratios or as counts and percentages, and associations are examined using the Pearson chi-squared test. Correlations between numerical variables are examined using Spearman’s rank correlation. Receiver-operating characteristic (ROC) curve analysis was used to examine the discriminative value of ADAM6 or PRSS1. *P-*Values < 0.05 are considered statistically significant.

#### Data acquisition and processing to identify ADAM6 and PRSS1 signatures in AML and ALL publicly available datasets

All required clinical and RNA-Seq data for both AML and ALL were obtained from The Genomic Data Commons (GDC) database (https://portal.gdc.cancer.gov/) and Therapeutically Applicable Research to Generate Effective Treatments (TARGET) via UCSC Xena platform (https://xena.ucsc.edu/). All transcriptomic data used for later analysis was in STAR Counts format. The retrieved datasets included AML samples (*n* = 3,698) and ALL samples (*n* = 997) of both normal and tumor origin where 60,660 genes are expressed across acute leukemia subtypes published in GDC and TARGET database. Next, preprocessing of TARGET data included merging RNA-Seq data for both AML and ALL, respectively, with corresponding clinical data based on similar TARGET sample IDs in “tissue_type.samples” category (“Tumor” or “Normal”). Both AML and ALL tumor samples were identified following samples with (“Primary Blood Derived Cancer—Bone Marrow”, “Primary Blood Derived Cancer – Peripheral Blood”, “Recurrent Blood Derived Cancer—Bone Marrow”, “Recurrent Blood Derived Cancer – Peripheral Blood”) subtypes in “sample_type.samples” category, while AML and ALL normal samples were identified for samples with (“Bone Marrow Normal”, “Blood Derived Normal”) subtypes in above mentioned sample group. The final preprocessed datasets for both TARGET AML and TARGET ALL transcriptomic data were then assayed for study of relevant gene expression levels of studied genes.

Next, a list of differentially expressed genes across both AML and ALL samples was generated. The R packages “ensembldb v. 2.28.1” and “AnnotationHub v.3.12.0” were used to convert Ensembl IDs for all genes listed in both TARGET AML and TARGET ALL gene expression data frames. Next, DEGs were calculated based on linear regression models through R package “limma v.3.60.4” based on specific criteria for selection; false-positive discovery (FDR) ≤ 0.05, *p-*Value ≤ 0.05; then log fold change was calculated using Benjamini–Hochberg (BH) method for P-adjusted scores for all DEGs. Lastly, using R package “ggplot2 v.3.5.1”, box plots for both ADAM6 and PRSS1 DEGs were generated.

## Results

### Clinical, demographic, and immunophenotyping characteristics of the studied groups.

The clinical, demographic, and immunophenotyping data of the studied groups are summarized in Table 1. Further details for the enrolled ALL and AML patients are provided in Supplementary Tables S1–S4.

**Table 1:** Clinical, demographic and immunophenotyping data of the studied groups

Groups/Parameters	Control group	Acute leukemia	ALL group	AML group
**N**	55	76	36	40
**Age (y)**	37(26–48)	33(25–44.5)	27.5(22–34.5)	37(26–48)
**TLC (k/mm3)**	-	16.5(3–60)*	34.5(8–165)*	10.3(2–35.5)*
**Hb (g/dl)**	-	7.45(6.6–8)*	7.3(6–8)*	7.4(7–8.2)*
**Plts (k/mm3)**	-	45(22–76)*	44(19.5–72)*	49(22.5–76)*
**LDH (IU/l)**	-	-	455(235–544)*	-
**Uric acid (mg/dl)**	-	-	7(5–8)*	-
**Blasts in BM after induction chemotherapy (%)**	-	-	-	3(2–9)*
**CD22 + **	-	-	15	–
**-**			21	–
**CD45 + **	-	-	15	–
**-**		-	21	–
**CD34 + **	-	-	-	26
**-**		-	-	14
**CD117 + **	-	-	-	32
**-**		-	-	8
**HLD-DR + **	-	38	4	34
**-**	-	38	32	6
**ADAM6 (pg/ml)**	78.6 (55.8–101.8)	207.9 (162.7–417.8)*	208.7 (178–337)*	186.4 (155–479)*
**PRSS1 (ng/ml)**	247.5 (204.3–375.3)	175.3 (151.2—217.9)*	175.1 (153.7—232.2)*	177.9 (145.3—206.4)*

Results are expressed as median and interquartile range (IQR)(25 th quartile-75 th quartile),* Significantly different from control (group I) at *p* > 0.0001

*N* Number of subjects in each group, *TLC* Total leukocyte count, *hb* hemoglobin, *plts* platelet, *LDH* Lactate dehydydrogenase, *CD* Cluster of differentiation, *HLA-Dr* Human leukocyte death receptor antigen

### Serum levels of ADAM6 in acute leukemia patients

**Fig. 1:**
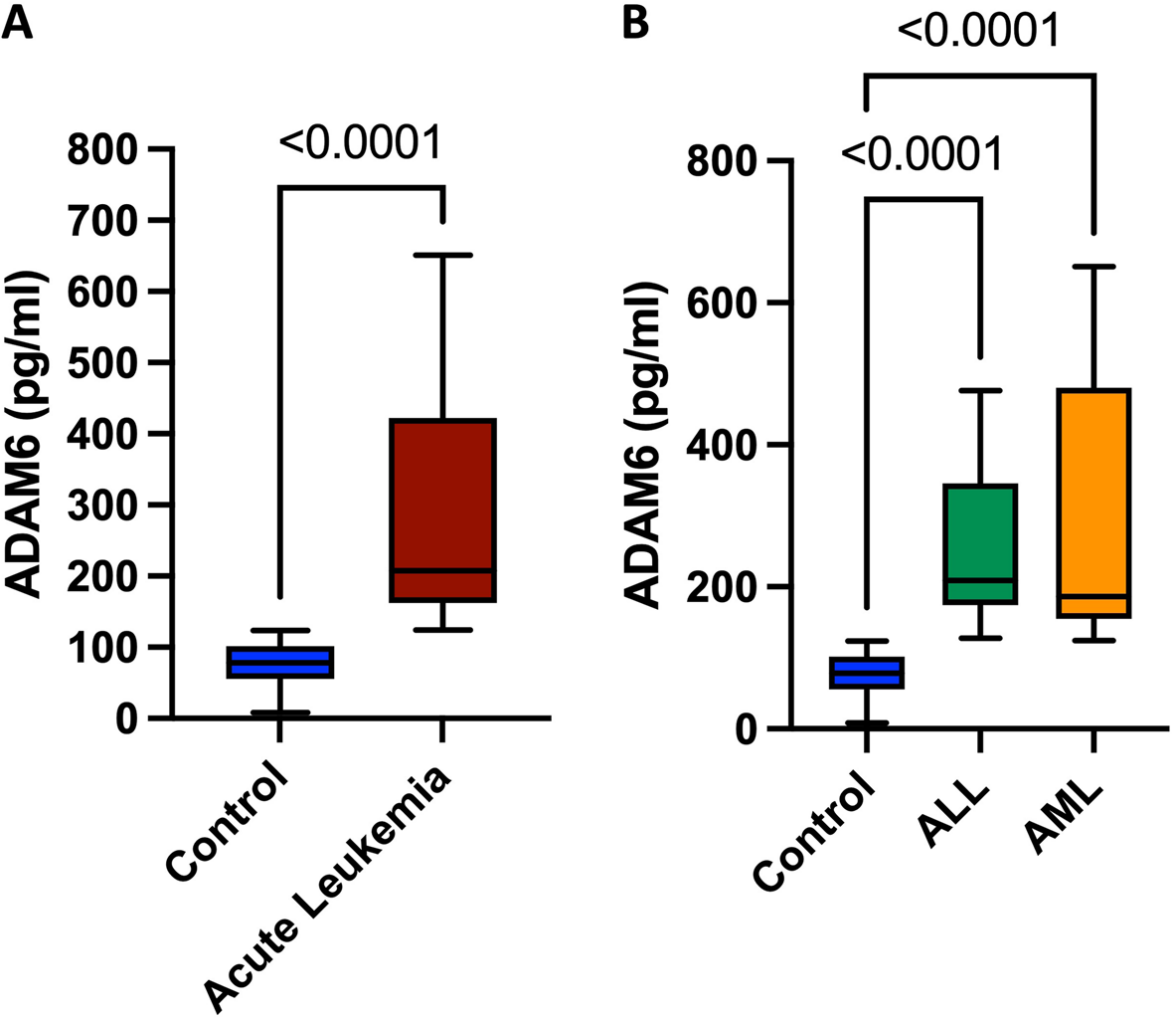
Serum levels of ADAM6 in acute leukemia patients. **A** ADAM6 levels in acute leukemia patients (*n* = 76) compared to healthy control subjects (*n* = 55). **B **ADAM6 levels in ALL patients (*n* = 36) and AML patients (*n* = 40) compared to healthy control subjects (*n* = 55). Box plots represent the interquartile range, the line inside the box represents the median, and the bars represent the minimum and maximum values. Shown *p-*Value*s* are for Kruskal–Wallis test. ALL, acute lymphoblastic leukemia; AML, acute myeloid leukemia; and ADAM6, A Disintegrin And Metalloproteinase Domain-6

Serum levels of ADAM6 were significantly higher in acute leukemia patients compared to control subjects (207.9 (162.7–417.8) and 78.6(55.8–101.8), *p* < *0.0001*), respectively, as shown in Fig. 1A. When acute leukemia patients were further stratified into ALL and AML groups, serum levels of ADAM6 were found to be significantly higher in both ALL and AML patients compared to control group (208.7 (178.3–337.3), 186.4 (155.3–479.6) and 78.6 (55.8–101.8) pg/ml, *p* < *0.0001*), respectively as shown in Fig. 1B. Additional data regarding the relation between ADAM6 and relevant disease characteristics in ALL, AML, or all acute leukemia patients are provided in Supplementary Table S5. It’s noteworthy here that no significant difference was found when comparing ADAM6 levels in ALL subtypes, T-ALL versus B-ALL patients (207.9 (173.5–391.0) and 211.1(170.3–320.0) pg/ml, *p* = *0.7022)* as shown in Supplementary Figure S3.

### Serum levels of PRSS1 in acute leukemia patients

**Fig. 2:**
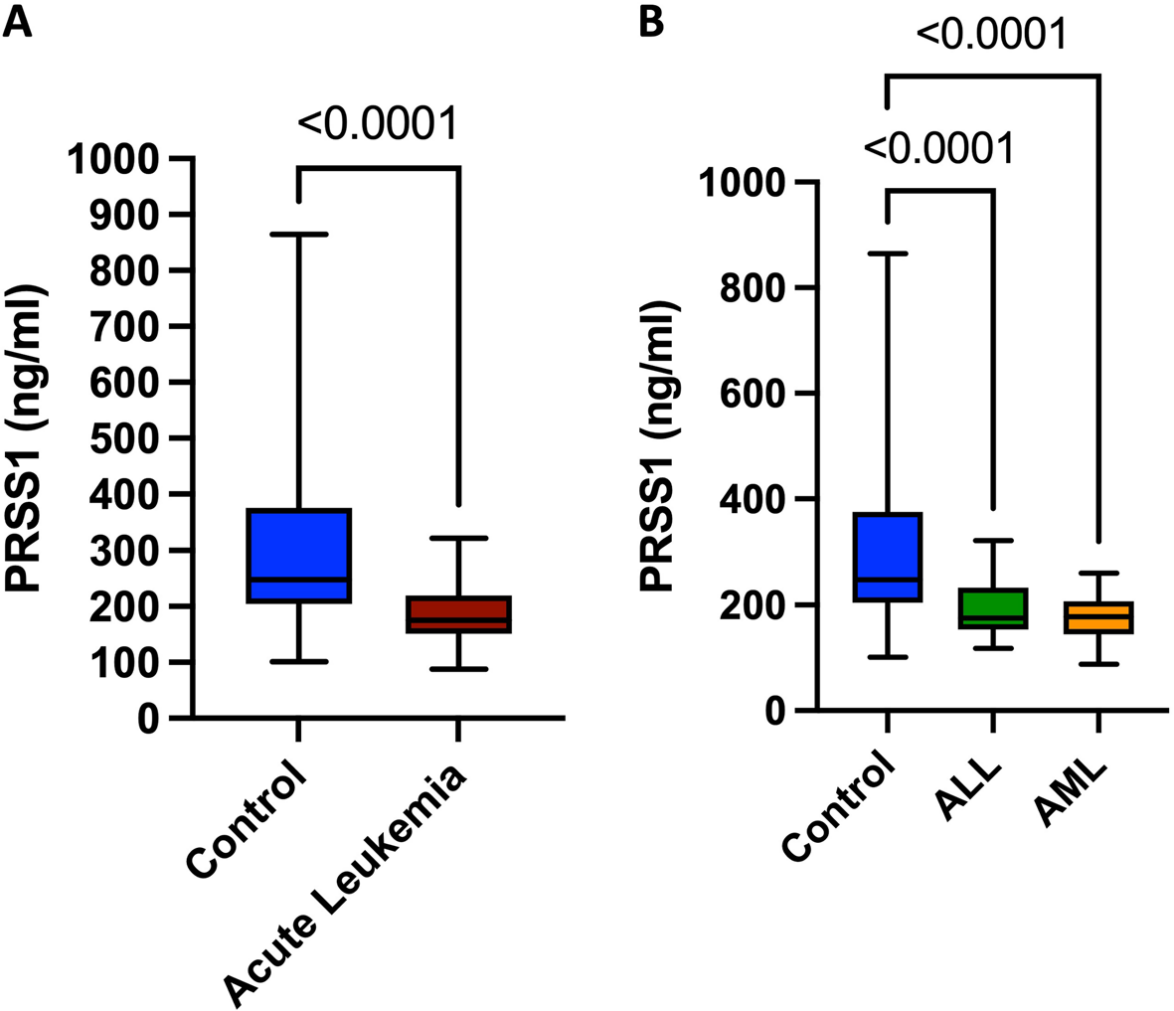
Serum levels of PRSS1 in acute leukemia patients. **A** PRSS1 levels in acute leukemia patients (*n* = 76) compared to healthy control subjects (*n* = 55). **B** PRSS1 levels in ALL patients (*n* = 36) and AML patients (*n* = 40) compared to healthy control subjects (*n* = 55). Box plots represent the interquartile range, the line inside the box represents the median, and the bars represent the minimum and maximum values. Shown *p-*Value*s* are for Kruskal–Wallis test. ALL, acute lymphoblastic leukemia; AML, acute myeloid leukemia; and PRSS1, Serine protease 1

Serum levels of PRSS1 were found to be significantly lower in acute leukemia patients compared to control subjects (175.3 (151.2–217.9) and 247.5 (204.3–375.3) ng/ml, *p* < *0.0001*), respectively, as shown in Fig. 2A. When acute leukemia patients were further stratified into ALL and AML groups, serum levels of PRSS1 were found to be significantly lower in ALL and AML patients compared to control group (175.1 (153.7–232.2), 177.9 (145.3–206.4) and 247.5 (204.3–375.3) ng/ml, *p* < *0.0001*), respectively as shown in Fig. 2B. Additional data regarding the relation between PRSS1 and relevant disease characteristics in ALL, AML, or all acute leukemia patients are provided in Supplementary Table S6. It’s noteworthy here that no significant difference was found when comparing PRSS1 levels in ALL subtypes, T-ALL versus B-ALL patients (206.1(158.6–239.3) and 175.1(151.6–230.3) ng/ml, *p* = *0.4687)* as shown in Supplementary Figure S3.

### The diagnostic significance of ADAM6 and PRSS1 using ROC analysis

**Fig. 3:**
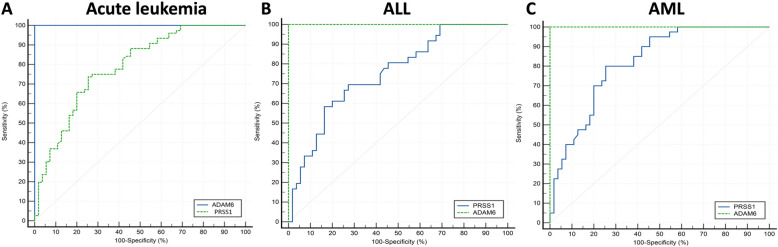
The diagnostic significance of ADAM6 and PRSS1 using ROC analysis. **A** ROC curves for discrimination between acute leukemia patients and healthy control subjects using ADAM6 or PRSS1. ADAM6 showed excellent diagnostic value (AUC = 1, sensitivity = 100%, specificity = 100%, cutoff criterion > 123.8 pg/ml and 95% CI (0.972–1), while PRSS1 ROC curve showed good diagnostic value (AUC = 0.79, sensitivity = 73.68%, specificity = 74.5%, cutoff criterion < 207.6 ng/ml and 95% CI (0.707—0.854)). **B** ROC curves for discrimination between ALL patients and healthy control subjects using ADAM6 or PRSS1. ROC curve for ADAM6 showed excellent diagnostic value (AUC = 1, sensitivity = 100%, specificity = 100%, cutoff criterion > 123.76 pg/ml and 95% CI (0.972–1)), while PRSS1 showed good diagnostic value (AUC = 0.752, sensitivity = 69%, specificity = 73%, cutoff criterion < 214.44 ng/ml and 95%CI (0.651–837)). **C** ROC curves for discrimination between AML patients and healthy control subjects using ADAM6 or PRSS1. ROC curve for ADAM6 showed excellent diagnostic value (AUC = 1, sensitivity = 100%, specificity = 100%, cutoff criterion > 123.8 pg/mL and 95% CI (0.962–1)), while ROC curve for PRSS1 showed very good diagnostic value (AUC = 0.819, sensitivity = 80%, specificity = 74.5%, cutoff criterion < 207.6 ng/mL and 95% CI (0.727—0.890)). ROC, receiver operator characteristic curve; AUC, area under the curve; CI, confidence interval; ALL, acute lymphoblastic leukemia; AML, acute myeloid leukemia; PRSS1, Serine protease 1; and ADAM6, A Disintegrin And Metalloproteinase Domain-6

Regarding diagnosis, ROC curve was used for discrimination between the studied groups and control group using ADAM6 or PRSS1. As for acute leukemia, ROC curve for ADAM6 showed excellent diagnostic value (AUC = 1, sensitivity = 100%, specificity = 100%, cut-off criterion > 123.8 pg/mL and 95% confidence interval (CI) (0.972–1). While PRSS1 ROC curve showed good diagnostic value (AUC = 0.79, sensitivity = 73.68%, specificity = 74.5%, cut off criterion < 207.6 ng/mL and 95% CI (0.707–0.854) as shown in Fig. 3A. Concerning ALL specifically, ROC curve for ADAM6 showed excellent diagnostic value (AUC = 1, sensitivity = 100%, specificity = 100% a cut-off criterion > 123.76 pg/ml and 95% CI (0.972–1).While PRSS1 showed good diagnostic value (AUC = 0.752, sensitivity = 69%, specificity = 73%, cutoff criterion < 214.44 ng/mL and 95%CI (0.651–837) as shown in Fig. 3B. Regarding AML, ROC curve for ADAM6 level showed excellent diagnostic value (AUC = 1, sensitivity = 100%, specificity = 100%, cutoff criterion > 123.8 pg/mL and 95% CI (0.962–1), while ROC curve for PRSS1 showed very good diagnostic value (AUC = 0.819, sensitivity = 80%, specificity = 74.5%, cutoff criterion < 207.6 ng/mL and 95% CI (0.727–0.890) as shown in Fig. 3C.

### The prognostic significance of ADAM6 and PRSS1 in acute leukemia patients

**Fig. 4:**
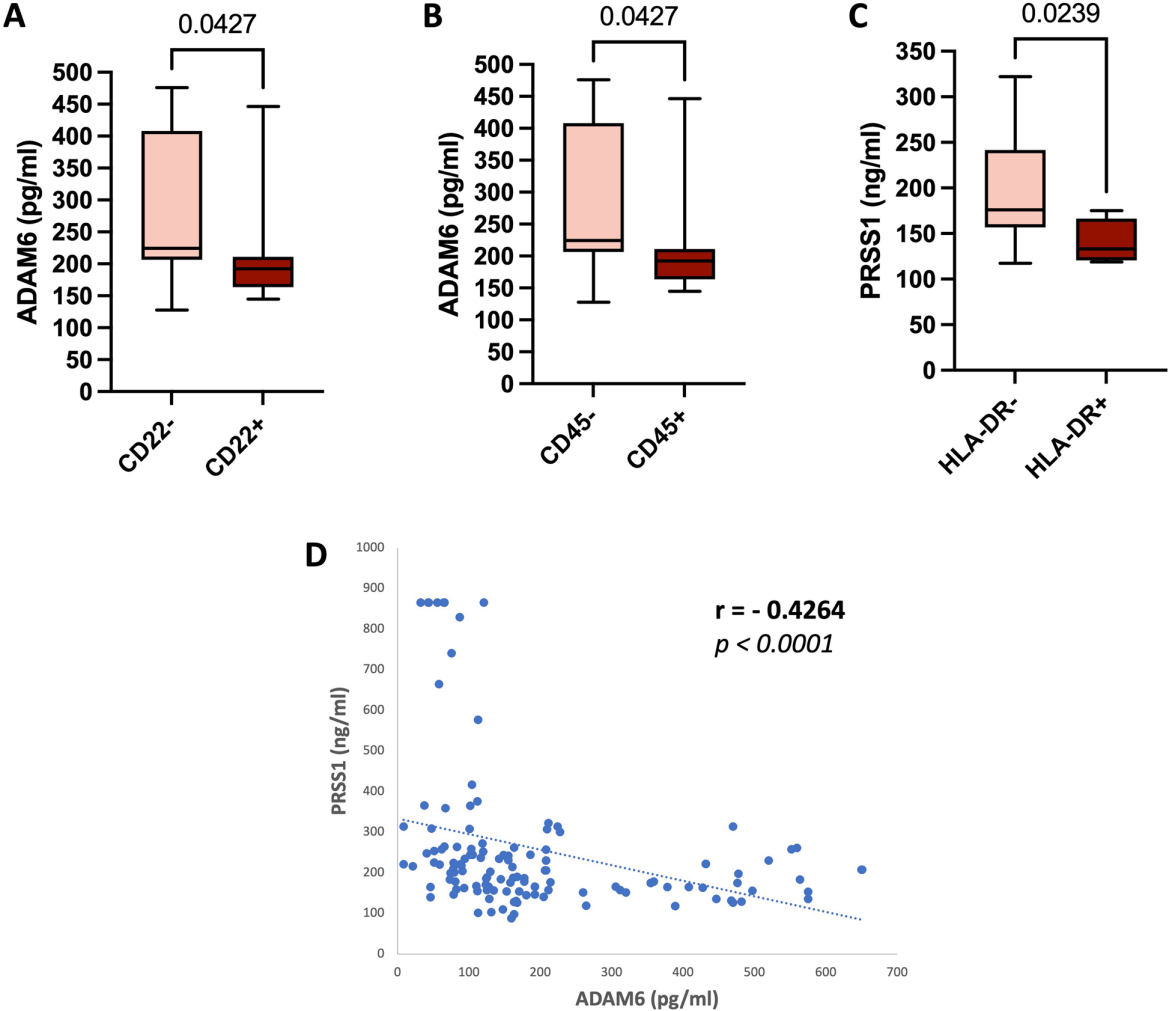
Association of ADAM6 and PRSS1 with classical prognostic markers in acute leukemia patients, and their correlation with each other. **A** Serum ADAM6 levels in CD22^−^ (*n* = 21) versus CD22^+^ (*n* = 15) ALL patients. **B** Serum ADAM6 levels in CD45^−^ (*n* = 21) versus CD45^+^ (*n* = 15) ALL patients. **C** Serum PRSS1 levels in HLA-DR^−^ (*n* = 32) versus HLA-DR^+^ (*n* = 4) ALL patients. **D** Spearman correlation analyses of serum ADAM6 and PRSS1 in all the study cohort, including acute leukemia patients and control subjects (*n* = 131). Box plots represent the interquartile range, the line inside the box represents the median, and the bars represent the minimum and maximum values. Shown *p-*Value*s* are for Mann–Whitney test. ALL, acute lymphoblastic leukemia; AML, acute myeloid leukemia; ADAM6, A Disintegrin And Metalloproteinase Domain-6; PRSS1, Serine protease 1; CD, cluster of differentiation; HLA-DR, human leucocyte antigen death receptor

Next, we attempted to investigate the relationship between serum levels of ADAM6 and PRSS1 with classical prognostic markers in ALL and AML patients. Interestingly, the serum levels of ADAM6 in CD22^+^ patients were found to be significantly lower compared to CD22^−^ ALL patients (*p* = *0.0427*, Fig. 4A) as well as being significantly lower in CD45^+^ patients as compared to CD45^−^ ALL patients (*p* = *0.0427*, Fig. 4B). Regarding PRSS1, its serum levels were found to be significantly lower in human leucocyte antigen death receptor (HLA-DR)^+^ compared to HLA-DR^−^ ALL patients (*p* = *0.0239*, Fig. 4C). However, no significant association was detected for ADAM6 or PRSS1, with HLA-DR and prognostic CDs in AML patients, as shown in Supplementary Tables S7 and S8.

### Correlations between serum levels of ADAM6 and PRSS1 and clinicopathological parameters in the studied groups

As shown in Table 2, serum levels of PRSS1 were found to be significantly negatively correlated with platelet count (*r* = − 0.353, *p* = *0.002*) in acute leukemia patients. Whereas, in ALL patients, serum levels of ADAM6 were found to be significantly negatively correlated with LDH (*r* = − 0.618, *p* = *0.018*), while serum levels of PRSS1 were found to be significantly negatively correlated with platelet count (*r* = − 0.370, *p* = *0.026*). Also, ADAM6 was found to be significantly positively associated with total leukocyte count (TLC) in AML patients (*r* = 0.382, *p* = *0.015*).

**Table 2:** Correlations between ADAM6 or PRSS1 and relevant quantitative variables in ALL, AML or all acute leukemia patients

		**ALL (*n* = 36)**		**AML (*n* = 40)**		**All acute leukemia patients (*n* = 76)**	
**Variable**		**ADAM6**	**PRSS1**	**ADAM6**	**PRSS1**	**ADAM6**	**PRSS1**
**Age**	Rho	−0.233	−0.146	0.055	−0.384*	−0.054	−0.292*
	*p-*Value	0.172	0.395	0.734	**0.014**	0.640	**0.011**
	N	36	36	40	40	76	76
**TLC**	Rho	−0.324	0.211	0.382*	0.105	0.139	0.182
	*p-*Value	0.054	0.216	**0.015**	0.519	0.232	0.115
	N	36	36	40	40	76	76
**Hemoglobin**	Rho	0.152	−0.257	0.131	−0.043	0.157	−0.137
	*p-*Value	0.375	0.130	0.419	0.793	0.176	0.239
	N	36	36	40	40	76	76
**Platelets**	Rho	0.132	−0.370*	−0.113	−0.299	−0.045	−0.353**
	*p-*Value	0.441	**0.026**	0.487	0.061	0.702	**0.002**
	N	36	36	40	40	76	76
**LDH**	Rho	−0.618*	−0.258	-	-	−0.618*	−0.258
	*p-*Value	**0.018**	0.374	-	-	**0.018**	0.374
	N	14	14	-	-	14	14
**ESR**	Rho	0.491	0.465	-	-	0.491	0.465
	*p-*Value	0.074	0.094	-	-	0.074	0.094
	N	14	14	-	-	14	14
**CRP**	Rho	−0.269	0.000	-	-	−0.269	0.000
	*p-*Value	0.353	1.000	-	-	0.353	1.000
	N	14	14	-	-	14	14
**Uric acid**	Rho	−0.125	0.180	-	-	−0.125	0.180
	*p-*Value	0.671	0.538	-	-	0.671	0.538
	N	14	14	-	-	14	14
**Blasts in BM aspirate**	Rho	0.179	−0.009	-	-	0.179	−0.009
	*p-*Value	0.296	0.960	-	-	0.296	0.960
	N	36	36	-	-	36	36
**Blasts in BM aspirate after induction chemotherapy**	Rho	-	-	0.634**	0.254	0.634**	0.254
	*p-*Value	-	-	**0.006**	0.325	**0.006**	0.325
	N	-	-	17	17	17	17
**Blasts in BM aspirate after 6 months**	Rho	-	-	−0.167	−0.058	−0.167	−0.058
	*p-*Value	-	-	0.604	0.857	0.604	0.857
	N	-	-	12	12	12	12
**MRD after induction chemotherapy**	Rho	-	-	0.341	0.082	0.341	0.082
	*p-*Value	-	-	0.181	0.756	0.181	0.756
	N	-	-	17	17	17	17
**MRD after 6 months**	Rho	-	-	−0.142	−0.204	−0.142	−0.204
	*p-*Value	-	-	0.696	0.571	0.696	0.571
	N	-	-	10	10	10	10
**ADAM6**	Rho	-	−0.211	-	0.225	-	0.066
	*p-*Value	-	0.218	-	0.163	-	0.574
	N	-	36	-	40	-	76
**PRSS1**	Rho	−0.211	-	0.225	-	0.066	-
	*p-*Value	0.218	-	0.163	-	0.574	-
	N	36	-	40	-	76	-

*N* Number, *Rho* Spearman’s rho

### Correlation between serum levels of ADAM6 and PRSS1

In the whole study cohort, including control subjects and acute leukemia patients, Serum levels of ADAM6 were found to be significantly negatively correlated with serum levels of PRSS1 (*r* = −0.4264, *p* < *0.0001*, Fig. 4D).

### Identifying ADAM6 and PRSS1 expression levels in AML and ALL publicly available datasets

During this study, TARGET AML (*n* = 3,698) and TARGET ALL (*n* = 997) samples were used for calculating gene expression levels of differentially expressed genes (DEGs) across both normal and acute leukemia samples. Using selected criteria of *p-*Value ≤ 0.05 and FDR ≤ 0.05, a total of 28,363 genes were differentially expressed in TARGET Acute Myeloid Leukemia samples out of all 60,660 genes assayed. Similarly, 33,469 genes were differentially expressed in TARGET Acute Lymphoblastic Leukemia samples out of all 60,660 genes assayed in the later dataset. ADAM6 was found to be significantly upregulated in TARGET AML tumor samples compared to normal (FC = 1.144, *p* ≤ *0.001*) (Supplementary Figure S4 A), while PRSS1 was found to be downregulated in TARGET AML tumor samples compared to normal samples (FC = −1.698, *p* ≤ *0.001*) (Supplementary Figure S4B). Also, in TARGET ALL samples, ADAM6 was found to be significantly upregulated in ALL tumor compared to normal (FC = 1.434, *p* ≤ *0.001*) (Supplementary Figure S4 C), while PRSS1 was significantly downregulated in ALL tumor against normal samples (FC = −1.515, *p* ≤ *0.001*) (Supplementary Figure S4D).

## Discussion

In the current study, we sought to determine the serum levels of ADAM6 and PRSS1, novel potential biomarkers with emerging implications in leukemogenesis, in adult patients with de novo acute leukemia compared to apparently healthy subjects. We also studied their potential as novel diagnostic/prognostic biomarkers for acute leukemia and its subtypes, ALL and AML. Interestingly, ADAM6 serum levels were found to be significantly higher while PRSS1 serum levels were found to be significantly lower in acute leukemia patients compared to their control counterparts. Moreover, serum ADAM6 showed significant variation between CD22^+^ and CD22^−^, as well as CD45^+^ and CD45^−^ ALL patients, while PRSS1 significantly varied between HLA-DR^+^ and HLA-DR^−^ ALL patients. These results portray both ADAM6 and PRSS1 as prominent novel biomarkers for both diagnosis and prognosis of adult acute leukemia patients.

In this study, ADAM6 serum levels were found to be significantly higher in both ALL and AML patients compared to healthy control subjects. This observation comes in agreement with the previously reported elevated expression levels of other members of the ADAM family in other cancer types, such as ADAM10/12/17 in early gastric cancer [45], ADAM8/9/15 in primary multiple myeloma [46] and ADAM12 in colorectal cancer [47] as well as breast and liver cancer [48]. Furthermore, this is consistent with the hypothesized role of ADAM6, like other ADAM family members, to serve in autocrine and paracrine transactivation of the epidermal growth factor receptor (EGFR); an oncogene acting as key player in cancer development and progression [49]. It’s noteworthy here that EGFR transactivation has been reported to play a role in acute leukemia development via activation of mitogen-activated extracellular signal-regulated kinase (MEK), Phosphatidylinositol-3-kinase (PI3 K), and Janus-activated kinase/signal transducer and activator of transcription (JAK/STAT) [50]. Additionally, EGF signaling is positively associated with cell proliferation, cell-cycle progression, as well as increased migration and metastasis [51]. Interestingly, upon TCGA data analysis for lung adenocarcinoma, Lifeng et al*.* identified the interrelation of ADAM6 with miRNA-mRNA-lncRNA network for competitive endogenous RNA (ceRNA) [52]. Additionally, Chiu et al., via genome-wide characterization of copy number aberrations in melanoma circulating tumor cells, identified ADAM6 as a potential novel biomarker for melanoma [53].

Next, we carried out ROC analysis to evaluate the diagnostic potential of ADAM6 in acute leukemia. ADAM6 showed excellent diagnostic value in acute leukemia patients and its subtypes ALL and AML. Afterwards, to gain insights into the prognostic potential of ADAM6 for acute leukemia, we classified both AML and ALL patients based on the presence/absence of characteristic classical prognostic CDs. First, serum levels of ADAM6 in CD22^+^ ALL patients were found to be significantly lower compared to their CD22^−^ counterparts. CD22 is generally regarded as a poor prognostic marker in acute leukemia [54]. It is a differentiation antigen expressed on the surface of B-lineage cells from the early progenitor stage of development [54] until before terminal differentiation into plasma cells, which are mostly CD22^−^ [55]. In response to cross-linking of the B-cell receptor, CD22 molecules are rapidly phosphorylated on tyrosine residues in its cytoplasmic domain, thereby generating phospho-tyrosine motifs which recruit intracellular effector proteins containing Src homology-2 containing domains and, via its interaction with these effector molecules, CD22 modulates signal transduction [56]. CD22 phosphorylation results in the recruitment of the negative regulatory tyrosine phosphatase, SHP-1 [57]. As mentioned earlier, ADAMs can mediate transactivation of EGFR [49], thus, they might also modulate the action of SHP-1, thereby affecting CD22 downstream signaling [58, 59].

Second, serum levels of ADAM6 were found to be significantly lower in CD45^+^ ALL patients compared to CD45^−^ patients. The transmembrane protein tyrosine phosphatase CD45 is expressed by all nucleated cells of hematopoietic origin [60, 61], and is generally regarded as a poor prognostic marker in acute leukemia [62, 63]. CD45 is a protein tyrosine phosphatase which dephosphorylates Src kinases involved in the regulation of several signal transduction pathways, including the granulocyte/macrophage colony-stimulating factor, which acts as a key player in the pathogenesis of leukemia generally, and AML in particular [64].

Third, in the current study, serum levels of ADAM6 in ALL patients were found to be significantly negatively correlated with LDH. High serum LDH levels are typically associated with a poor prognosis in many cancer types, including acute leukemia [65]. LDH contributes to the enhanced glycolytic flux [66], thereby the Warburg effect which plays a major role in cancer progression [67]. It’s noteworthy here that our findings regarding the potential prognostic value of ADAM6, at least to some extent, corroborate the previous findings by Alsuwaidi and coworkers who reported the association of *ADAM6* gene homozygous deletions with a poor ten years of overall patients’ survival in B-ALL patients [20]. Thus, overall, ADAM6 can be regarded as a relatively good diagnostic biomarker in acute leukemia patients with a potential prognostic implication in ALL which requires further investigations in larger cohorts to be fully elucidated.

Regarding our second potential biomarker of interest, PRSS1, its serum levels were found to be significantly lower in both ALL and AML patients compared to healthy controls. It’s important to point here that trypsinogen and chymotrypsinogen have been reported to exhibit potent anti-tumor effects through various mechanisms such as: inducing redifferentiation of tumor cells, reducing cancer stem cells population via impairing their pluripotency and metastatic capacity, as well as affecting TGF-Beta pathway and protease-activated-receptors (PARs) [68]. This, at least partially, helps to explain the observed altered levels of PRSS1 in acute leukemia patients compared to healthy controls in our current study. Nevertheless, silencing of PRSS1 has been reported to inhibit the extracellular signal-regulated kinase (ERK) signaling pathway by reducing PAR-2 activation, thereby suppressing the growth and proliferation of gastric carcinoma cells [69]. It's noteworthy here that mutations in PRSS1 gene have been reported to possibly cause increased intra-acinar trypsin levels, thereby increasing the risk of auto-digestion leading to pancreatic inflammation, pancreatitis, and increased risk of pancreatic cancer [34]. Pancreatic secretory trypsin inhibitor (PSTI), also known as serine protease inhibitor Kazal type 1 (SPINK1), was suggested to be the first line of defense against premature trypsin activation in the pancreas since it inhibits PRSS1 [70]. Interestingly, raised SPINK1 levels have been reported to play a role in angiogenesis and trans endothelial migration of ALL cells due to the activation of various signaling pathways, including mitogen-activated protein kinases (MAPK)/ERK and PI3 K/AKT pathways [71].

In ROC analysis, PRSS1 showed good diagnostic value in acute leukemia patients and in ALL patients, and very good diagnostic value in AML patients, nominating it as a promising diagnostic biomarker for acute leukemias, especially AML. Furthermore, serum levels of PRSS1 were found to be significantly lower in HLA-DR^+^ compared to HLA-DR^−^ ALL patients. HLA-DR is a well-established poor prognostic marker [72]. The expression of HLA-DR has been shown to be an independent predictor of failure to achieve complete remission, and the absence of HLA-DR expression is known to be associated with good prognosis [72]. As mentioned earlier, PRSS1 increases trypsin activation, which is a proteolytic enzyme that can cleave proteins at specific sites. This might affect the processing of antigens before they are presented by HLA-DR; trypsin might digest proteins that would otherwise bind to HLA-DR, thereby leading to a possibly reduced presentation of HLA-DR.

Furthermore, serum levels of PRSS1 exhibited a significant negative correlation with platelet count in acute leukemia, specifically in ALL. When acute leukemia patients have low platelet count, this is considered a sign of poor prognosis [73, 74]. Our results are in agreement with the previous findings of Levavi and coworkers, who reported that *PRSS1* loss may play a role in leukemogenesis, and that patients with *PRSS1* loss were significantly less likely to achieve minimal residual disease (MRD) negativity with poorer treatment outcomes after induction [38]. Collectively, the findings of our study, together with all those previous reports, shed light on the potential complex in-context dependent role of PRSS1 in the development and progression of cancer generally, and leukemia particularly.

Finally, we attempted to investigate the possible interrelation between ADAM6 and PRSS1. Interestingly, ADAM6 and PRSS1 were found to be significantly negatively correlated with each other in the whole study cohort, including controls and acute leukemia patients. However, when we further studied their association within separate groups, they showed a tendency for a positive correlation in AML group, while a tendency for a negative correlation in the ALL and control groups, yet all these associations were statistically non-significant, as shown in Supplementary Figure S1. Afterwards, we further investigated their co-expression through analysis of pan-cancer lymphoid neoplasm diffuse large B-cells and AML datasets on STARBASE-ENCORI [75], in which they exhibited a significant positive association with each other as shown in Supplementary Figure S2.

It’s important to point here that previously Alsuwaidi and coworkers identified both *ADAM6* and *PRSS1* among the genes with the highest percentage of chromosomal changes in profiled B-cell precursor ALL patients, and identified PRSS1 among the potential novel players mediating *ADAM6* gene homodeletion effects in pediatric ALL [20]. Furthermore, PRSS1 has been identified as a key player in TME modulation which impacts high-grade serous ovarian carcinoma prognosis [39], and together with other genes within an ECM panel predicted by artificial intelligence can help to provide an accurate tool to assess the patient’s response to immunotherapy and forecast ovarian cancer prognosis [40]. On the other hand, ADAM6 has been reported to mediate the interaction of sperm with ECM and to aid sperm transport through the female reproductive tract with profound effects on male fertility [41].

Collectively, these previous reports shed light on the role of both ADAM6 and PRSS1 in mediating various interactions with ECM, thereby possibly modulating the TME. It’s important to point here that ECM is generally highly dynamic and several ECM components are continuously degraded, deposited, or modified, resulting in remodeling, which greatly impacts several biological processes, including cell differentiation, proliferation, and stem cell niche [76, 77]. Thus, improper ECM remodeling and altered tissue dynamics can ultimately lead to pathological consequences like cancer, and TME has indeed attracted much interest lately [78, 79]. These observations, together with the findings of the current study, highlight the potential in-context dependent interplay between ADAM6 and PRSS1. Furthermore, given the fact that the expression of CD surface markers like CD22, CD45, and HLA-DR is generally interrelated with the lineage and differentiation stage of immune hematopoietic cells [80–83], together with the observed significant variation of ADAM6 and/or PRSS1 levels within CD22^+^/CD22^−^, CD45^+^/CD45^−^, and HLA-DR^+^/HLA-DR^−^ ALL patients, these findings shed light on the possible interplay between ADAM6 and/or PRSS1 with the differentiation process of immune hematopoietic cells. A notion that requires further thorough high-throughput investigations to be confirmed.

It’s important to remember here that despite the function of ADAM6 remaining largely elusive, it still harbors a metalloproteinase domain [16, 49], which might act on PRSS1, either activating or inhibiting its function. Also, PRSS1 is a serine protease which might interact with ADAM6 one way or another, or act on its pro-domain moiety, thereby affecting its activity. Besides, ADAMs have been generally implicated in growth factors and receptors [17, 46] and emerged as key therapeutic targets to inhibit cancer initiation and progression [84]. PRSS1 has also been reported to modulate several PARs, which are key players in cancer [34, 69]. Thus, both ADAM6 and PRSS1 can modulate several signaling cascades interrelated with leukemogenesis and cancer progression.

Furthermore, it’s important to point here that upon analysis of publicly available TARGET datasets for much larger cohorts of AML and ALL; ADAM6 and PRSS1 exhibited the same observed pattern of differential expression in acute leukemia patients compared to normal controls. ADAM6 was found to be significantly upregulated while PRSS1 was found to be downregulated in TARGET AML tumor samples compared to normal samples. Likewise, ADAM6 was found to be significantly upregulated while PRSS1 was significantly downregulated in TARGET ALL tumor compared to normal samples as shown in Supplementary Figure S4.

Thus, the findings of the current study open the door for future mechanistic studies to investigate ADAM6 and PRSS1 in various cancer types, including both hematological malignancies and solid tumors, and shed light on the need for careful consideration of various molecular mediators interrelated with TME remodeling. Nevertheless, further investigations are indeed warranted to elucidate the molecular mechanisms of this interplay and its implication in the pathogenesis and progression of cancer generally, and acute leukemia specifically. Also, future studies are warranted to investigate the possible interplay between PRSS1 and other ADAM family members in acute leukemia, as well as various cancer types.

Limitations of the current study include the lack of data regarding the relationship of ADAM6 and/or PRSS1 levels with overall survival (OS) and relapse-free survival (RFS), as well as not providing in-depth profiling for ADMA6 and/or PRSS1 in different genetic subtypes of AML or ALL. Additionally, the current study adopted a cross-sectional design in which the levels of ADAM6 and PRSS1 were measured in patients with acute leukemia and control subjects at one time point and cannot detect a cause-effect relationship. However, the findings of the current study provide a proof-of-principle, opening the door for future large-scale clinical studies to further elucidate the role of ADAM6 and/or PRSS1 in acute leukemia, as well as other types of cancer. Such studies can undoubtedly further elucidate their potential as novel therapeutic targets in leukemia and cancer in general.

## Conclusion

To the best of our knowledge, this is the first study to investigate the role ADAM6 and PRSS1 in adult patients with acute leukemia, namely ALL and AML. Higher circulating ADAM6 and lower circulating PRSS1 levels were observed in acute leukemia patients compared to healthy control subjects. Both ADAM6 and PRSS1 exhibited a strong potential as novel diagnostic biomarkers for acute leukemia. Furthermore, they were found to be associated with some classical prognostic biomarkers like CD22, CD45 and HLA-DR in ALL, which sheds light on their prognostic potential. Moreover, ADAM6 and PRSS1 showed a tendency for in-context dependent association with each other. Conclusively, the results of the current study portray ADAM6 and PRSS1 as potential novel diagnostic/prognostic biomarkers and therapeutic targets in acute leukemia. Further, in-depth thorough molecular studies are warranted to elucidate the mechanism of action of ADAM6 and PRSS1 in acute leukemia, and also to study their possible role and interrelation in other hematological malignancies and solid tumors. Unravelling the molecular basis of their interplay with each other, as well as each of them with other interrelated molecular mediators, can provide a myriad of novel potential therapeutic targets for adult patients with AML or ALL.

## Supplementary Information

Supplementary Material 1.

## Data Availability

“Data is provided within the manuscript or supplementary information files”.

## Abbreviations

ADAM: A Disintegrin And Metalloproteinase
ALL: Acute lymphoblastic leukemia
AML: Acute myeloid leukemia
CD: Cluster of differentiation
ECM: Extracellular matrix
ELISA: Enzyme linked immunosorbent assay
ERK: Extracellular signal-regulated kinase
Hgb: Hemoglobin
HLA-DR: Human leukocyte antigen-DR
JAK: Janus-activated kinase
LDH: Lactate dehydrogenase
MAPK: Mitogen-activated protein kinases
MEK: Mitogen-activated extracellular signal-regulated kinase
PARs: Protease activated receptors
PI3 K: Phosphatidylinositol-3-kinase
PLT: Platelets
PRSS1: Serine protease 1
PSTI: Pancreatic secretory trypsin inhibitor
ROC: Reciever operating characteristic
STAT: Signal transducer and activator of transcription
SPINK1: Serine protease inhibitor Kazal type1
TME: Tumor microenvironment
TLC: Total leukocyte count
