# Supplementary material for: Emerging roles of ADAM6 and PRSS1 as novel diagnostic/prognostic biomarkers for acute lymphoblastic and myeloid leukemia in adults
Source: BMC Cancer. 2025 May 17;25:884. doi: 10.1186/s12885-025-14292-9 (PMC12085065; doi:10.1186/s12885-025-14292-9)
Supplement: Supplementary file 1 — Supplementary Material 1. [file 12885_2025_14292_MOESM1_ESM.pdf]

Supplementary Table S1: Disease characteristics of ALL patients - Categorical variables

Variable		Count	Valid %
**Subtype**	Pre-B	17	47.2%
	Pro-B	6	16.7%
	T	13	36.1%
**Sex**	F	20	55.6%
	M	16	44.4%
**Biochemical work-up**	Normal	36	100.0%
	High serum creatinine (1.8 mg/dl)	0	0.0%
**CD2**	-	29	80.6%
	+	7	19.4%
**cytCD3**	-	34	94.4%
	+	2	5.6%
**CD4**	-	33	91.7%
	+	3	8.3%
**CD5**	-	30	83.3%
	+	6	16.7%
**CD7**	-	30	83.3%
	+	6	16.7%
**CD8**	-	31	86.1%
	+	5	13.9%
**CD10**	-	10	27.8%
	+	26	72.2%
**CD19**	-	9	25.0%
	+	27	75.0%
**CD20**	-	33	91.7%
	+	3	8.3%
**CD22**	-	21	58.3%
	+	15	41.7%
**CD33**	-	35	97.2%
	+	1	2.8%
**CD34**	-	29	80.6%
	+	7	19.4%
**CD45**	-	21	58.3%

	+	15	41.7%
**CD56**	-	20	55.6%
	+	16	44.4%
**CD58**	-	30	83.3%
	+	6	16.7%
**CD79A**	-	28	77.8%
	+	8	22.2%
**TDT**	-	14	38.9%
	+	22	61.1%
**SCD3**	-	35	97.2%
	+	1	2.8%
**TCR-AB**	-	35	97.2%
	+	1	2.8%
**HLA-DR**	-	32	88.9%
	+	4	11.1%
**t(9;22)**	-	8	57.1%
	+	6	42.9%
**CytoCONV**	46xx	8	22.2%
	46xy	28	77.8%
**Intramedullary infiltration**	-	36	100.0%
	+	0	0.0%
**CNS infiltration**	-	26	72.2%
	+	10	27.8%
**Blasts in CSF**	-	12	85.7%
	+	2	14.3%
**Risk stratification**	Low	2	5.6%
	Standard	15	41.7%
	High	19	52.8%
**Response to chemotherapy**	Complete remission	12	33.3%
	Resistant	3	8.3%
	Died	21	58.3%

Supplementary Table S2: Disease characteristics of ALL patients - Numerical variables

						Percentile			
Variable	Valid N	Mean	SD	Min.	Max.	25th	50th	75th	Normality test†
Age (yr)	36	28.4	8.3	16.0	45.0	22.0	27.5	34.0	<0.0001
TLC (k/mm^3)	36	111.1	150.7	2.0	560.0	8.0	34.5	165.0	<0.0001
Hemoglobin (g/dl)	36	7.6	2.2	4.0	16.0	6.0	7.3	8.0	<0.0001
Platelets (k/mm^3)	36	50.0	35.7	3.0	150.0	19.5	44.0	72.5	<0.0001
LDH (IU/l)	14	394	188	2	653	235	455	544	<0.0001
ESR (mm/h)	14	114	61	33	254	77	106	122	<0.0001
CRP (mg/l)	14	5	2	2	9	3	5	8	<0.0001
Uric acid (mg/dl)	14	6	2	3	9	5	7	8	<0.0001
Blasts in BM aspirate (%)	36	82	17	34	98	80	90	90	<0.0001
ADAM6(pg/ml)	36	256.9	110.5	127.5	476.0	178.3	208.7	337.3	<0.0001
PRSS1 (ng/ml)	36	195.3	60.7	117.5	322.0	153.7	175.1	232.2	<0.0001

†. D'Agostino-Pearson test.

SD = standard deviation, Min. = minimum, Max. = maximum.

Supplementary Table S3: Disease characteristics of AML patients - Categorical variables

Variable		Count	Valid %
	M0	1	2.5%
	M0-M1	1	2.5%
	M1	5	12.5%
	M1-M2	6	15.0%
	M2	12	30.0%
	M3	3	7.5%
	M4	8	20.0%
	M5	4	10.0%
**Sex**	F	15	37.5%
	M	25	62.5%
**Biochemical work-up**	Normal	39	97.5%
	High serum creatinine (1.8 mg/dl)	1	2.5%
**CD4**	-	39	97.5%
	+	1	2.5%
**CD7**	-	38	95.0%
	+	2	5.0%
**CD10**	-	39	97.5%
	+	1	2.5%
**CD11c**	-	39	97.5%
	+	1	2.5%
**CD13**	-	4	10.0%
	+	36	90.0%
**CD19**	-	39	97.5%
	+	1	2.5%
**CD30**	-	39	97.5%
	+	1	2.5%
**CD33**	-	5	12.5%
	+	35	87.5%
**CD34**	-	14	35.0%
	+	26	65.0%
**CD45**	-	36	90.0%
	+	4	10.0%

**CD56**	-	39	97.5%
	+	1	2.5%
**CD58**	-	39	97.5%
	+	1	2.5%
**CD64**	-	16	40.0%
	+	24	60.0%
**CD117**	-	8	20.0%
	+	32	80.0%
**MPO**	-	11	27.5%
	+	29	72.5%
**HLA-DR**	-	6	15.0%
	+	34	85.0%
**t(8;21)**	-	39	97.5%
	+	1	2.5%
**t(16;16)**	-	2	100.0%
	+	0	0.0%
**t(9;22)**	-	0	0.0%
	+	2	100.0%
**CytoCONV**	46xx	23	57.5%
	46xy	17	42.5%
**Response to chemotherapy**	Complete remission	16	40.0%
	Resistant	8	20.0%
	Died	16	40.0%

Supplementary Table S4: Disease characteristics of AML patients - Numerical variables

						Percentile			
variable	Valid N	Mean	SD	Min.	Max.	25th	50th	75th	Normality test†
**Age (yr)**	40	40.7	12.3	21.0	60.0	29.5	40.5	51.5	*<0.0001*
**TLC (k/mm^3)**	40	26.6	42.3	1.0	186.0	2.0	10.3	35.5	*<0.0001*
**Hemoglobin (g/dl)**	40	7.5	1.5	2.9	10.9	7.0	7.4	8.2	*<0.0001*
**Platelets (k/mm^3)**	40	51.6	34.7	5.0	150.0	22.5	49.5	76.0	*<0.0001*
**Blasts in BM after induction chemotherapy (%)**	17	10.3	18.2	1.0	74.0	2.0	3.0	9.0	*<0.0001*
**Blasts in BM after 6 months (%)**	12	1.8	1.5	1.0	6.0	1.0	1.0	2.0	*<0.0001*
**MRD after induction chemotherapy (%)**	17	8.8	18.5	0.0	72.0	0.0	0.2	9.0	*<0.0001*
**MRD after 6 months (%)**	10	1.0	3.2	0.0	10.0	0.0	0.0	0.0	*<0.0001*
**ADAM6 (pg/ml)**	40	302.5	180.9	124.0	650.8	155.3	186.4	479.6	*<0.0001*
**PRSS1 (ng/ml)**	40	175.3	44.1	88.1	260.6	145.3	177.9	206.4	*<0.0001*

†. D'Agostino-Pearson test.

SD = standard deviation, Min. = minimum, Max. = maximum.

Supplementary Table S5: Relation between ADAM6 and relevant disease characteristics in ALL, AML or all acute leukemia patients

				ADAM6 (pg/ml)			
**Group**	**Variable**	**Category**	**N**	**Median**	**Q1**	**Q3**	***p-Value***
**ALL**	CNS infiltration	-	26	208.7	163.4	320.0	*0.502†*
		+	10	211.1	192.3	427.5	
	Blasts in CSF	-	12	178.3	160.7	219.4	*0.647†*
		+	2	200.1	192.3	207.9	
	Risk stratification	Low	2	190.1	152.6	227.6	*0.117‡*
		Standard	15	264.0	207.9	467.5	
		High	19	207.9	163.4	211.1	
	Response to chemotherapy	Complete remission	12	219.4	200.1	337.4	*0.605‡*
		Resistant	3	170.3	152.6	389.0	
		Died	21	207.9	186.3	312.0	
**AML**	CNS infiltration	-	-	-	-	-	-
		+	-	-	-	-	
	Blasts in CSF	-	-	-	-	-	-
		+	-	-	-	-	
	Risk stratification	Low	-	-	-	-	-
		Standard	-	-	-	-	
		High	-	-	-	-	
	Response to chemotherapy	Complete remission	16	192.2	147.1	495.6	*0.406‡*
		Resistant	8	158.6	129.9	174.0	

		Died	16	368.4	171.8	489.4	
**All acute leukemias**	CNS infiltration	-	26	208.7	163.4	320.0	*0.502†*
		+	10	211.1	192.3	427.5	
	Blasts in CSF	-	12	178.3	160.7	219.4	*0.647†*
		+	2	200.1	192.3	207.9	
	Risk stratification	Low	2	190.1	152.6	227.6	*0.117‡*
		Standard	15	264.0	207.9	467.5	
		High	19	207.9	163.4	211.1	
	Response to chemotherapy	Complete remission	28	209.5	162.0	419.8	*0.490‡*
		Resistant	11	162.0	131.1	180.4	
		Died	37	211.1	177.5	431.6	

†. Mann-Whitney test

‡. Jonckheere-Terpstra test.

Q1 = 1st quartile, Q3 = 3rd quartile.

Supplementary Table S6: Relation between PRSS1 and relevant disease characteristics in ALL, AML or all acute leukemia patients

				PRSS1 (ng/ml)			
**Group**	**Variable**	**Category**	**N**	**Median**	**Q1**	**Q3**	***p-Value***
ALL	CNS infiltration	-	26	179.6	151.6	244.3	*0.584†*
		+	10	165.2	163.4	176.7	
	Blasts in CSF	-	12	175.3	153.7	225.2	*0.715†*
		+	2	197.7	165.2	230.3	
	Risk stratification	Low	2	226.9	153.8	300.0	*0.840‡*
		Standard	15	165.2	140.2	257.0	
		High	19	175.5	157.2	230.3	
	Response to chemotherapy	Complete remission	12	190.6	154.4	232.2	*0.562‡*
		Resistant	3	153.6	117.5	153.8	
		Died	21	175.5	163.4	244.3	
**AML**	CNS infiltration	-	-	-	-	-	-
		+	-	-	-	-	
	Blasts in CSF	-	-	-	-	-	-
		+	-	-	-	-	
	Risk stratification	Low	-	-	-	-	-
		Standard	-	-	-	-	
		High	-	-	-	-	
	Response to chemotherapy	Complete remission	16	185.6	156.9	206.4	*0.821‡*
		Resistant	8	148.6	130.5	186.2	

		Died	16	171.7	140.8	225.7	
**All acute leukemias**	CNS infiltration	-	26	179.6	151.6	244.3	*0.584†*
		+	10	165.2	163.4	176.7	
	Blasts in CSF	-	12	175.3	153.7	225.2	*0.715†*
		+	2	197.7	165.2	230.3	
	Risk stratification	Low	2	226.9	153.8	300.0	*0.840‡*
		Standard	15	165.2	140.2	257.0	
		High	19	175.5	157.2	230.3	
	Response to chemotherapy	Complete remission	28	185.6	156.9	211.0	*0.824‡*
		Resistant	11	152.9	125.8	185.3	
		Died	37	175.5	155.2	230.3	

†. Mann-Whitney test

‡. Jonckheere-Terpstra test.

N = number, Q1 = 1st quartile, Q3 = 3rd quartile.

Supplementary Table S7: Relation between ADAM6 or PRSS1 and relevant CDs in ALL patients

		ADAM6 (pg/ml)					PRSS1 (ng/ml)				
**Variable**		**N**	**Median**	**Q1**	**Q3**	***p-Value†***	**N**	**Median**	**Q1**	**Q3**	***p-Value†***
**CD10**	-	10	216.9	207.9	427.5	*0.358*	10	232.2	163.4	307.0	*0.210*
	+	26	207.9	163.4	320.0		26	175.1	153.6	206.1	
**CD19**	-	9	209.5	207.9	312.0	*0.687*	9	230.3	163.4	257.0	*0.289*
	+	27	207.9	163.4	354.5		27	175.1	151.6	214.4	
**CD22**	-	21	224.2	207.9	389.0	***0.043***	21	175.1	151.6	230.3	*0.596*
	+	15	192.3	163.4	211.1		15	175.5	153.8	244.3	
**CD34**	-	29	207.9	170.3	354.5	*0.603*	29	175.5	157.2	234.2	*0.223*
	+	7	214.3	204.7	320.0		7	156.8	140.2	176.7	
**CD45**	-	21	224.2	207.9	389.0	***0.043***	21	175.1	151.6	230.3	*0.596*
	+	15	192.3	163.4	211.1		15	175.5	153.8	244.3	
**CD56**	-	20	239.2	206.3	408.3	*0.062*	20	170.1	145.9	222.3	*0.293*
	+	16	200.1	163.4	217.7		16	179.6	155.5	253.1	
**CD79A**	-	28	207.9	166.9	245.8	*0.148*	28	175.3	155.5	239.3	*0.481*
	+	8	316.0	206.3	412.4		8	165.9	145.9	210.2	
**HLA-DR**	-	32	207.9	165.1	318.0	*0.176*	32	176.1	156.9	241.8	***0.024***
	+	4	309.4	219.5	441.2		4	133.1	120.6	166.3	

†. Mann-Whitney test

N = number, Q1 = 1st quartile, Q3 = 3rd quartile

Supplementary Table S8: Relation between ADAM6 or PRSS1 and relevant CDs in AML patients

		ADAM6 (pg/ml)					PRSS1 (ng/ml)				
**Variable**		**N**	**Median**	**Q1**	**Q3**	***p-Value†***	**N**	**Median**	**Q1**	**Q3**	***p-Value†***
**CD13**	-	4	294.2	157.6	483.8	*0.752*	4	160.5	150.4	212.6	*1.000*
	+	36	184.9	155.3	479.6		36	180.5	140.8	206.4	
**CD33**	-	5	378.0	180.4	481.6	*0.526*	5	144.3	128.9	164.8	*0.297*
	+	35	177.5	155.3	477.6		35	182.9	150.9	207.6	
**CD34**	-	14	193.6	134.8	551.6	*0.843*	14	190.0	144.3	207.6	*0.435*
	+	26	184.9	159.3	431.6		26	165.2	146.3	189.5	
**CD64**	-	16	309.4	156.2	479.6	*0.534*	16	160.6	139.8	199.8	*0.384*
	+	24	167.5	155.3	475.8		24	185.9	149.6	214.5	
**CD117**	-	8	156.2	130.6	370.0	*0.335*	8	167.3	139.8	205.1	*0.839*
	+	32	233.4	160.7	479.6		32	180.3	148.6	206.4	
**HLA-DR**	-	6	366.0	170.0	582.4	*0.228*	6	192.8	135.5	258.3	*0.409*
	+	34	184.9	153.6	443.1		34	171.7	143.5	203.2	

†. Mann-Whitney test

N = number, Q1 = 1st quartile, Q3 = 3rd quartile

Supplementary Figure S1

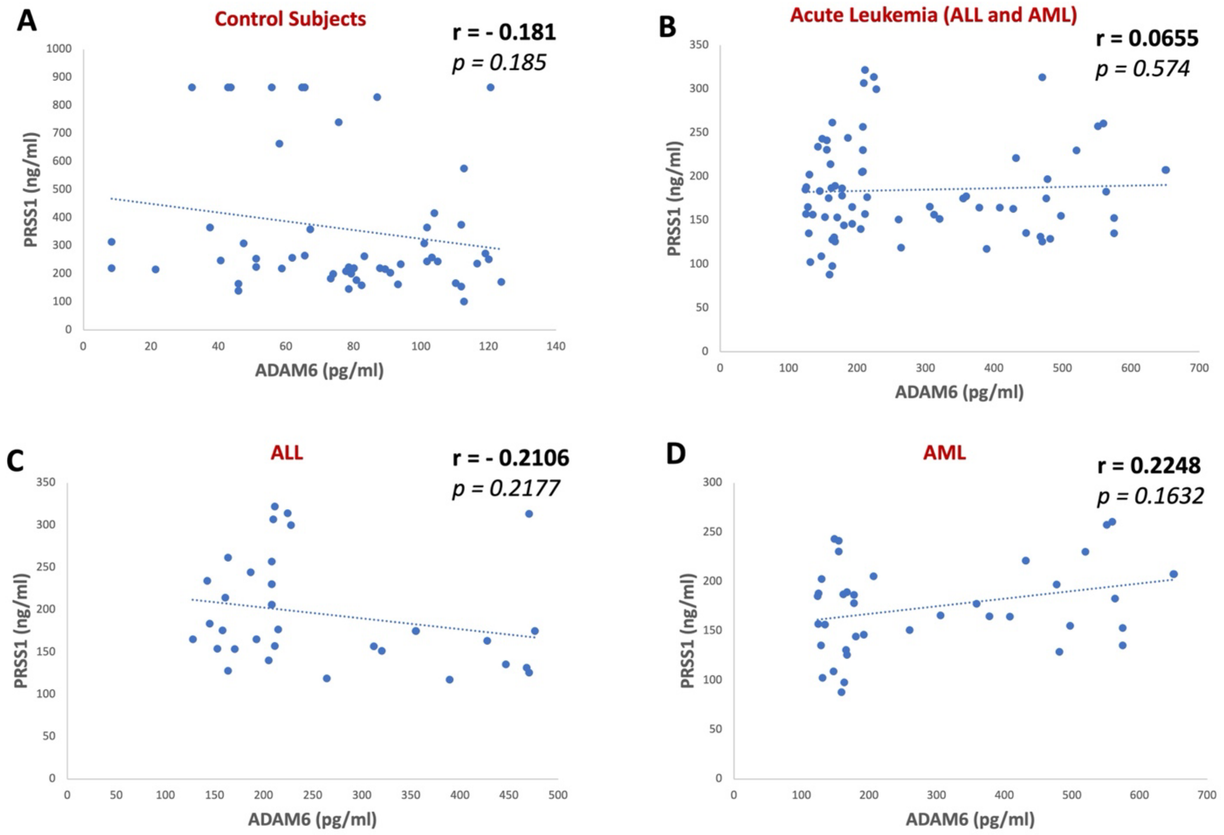

Spearman's rank correlation analyses between ADAM6 and PRSS1 in: (A) Control healthy subjects (n=55), (B) Acute leukemia patients (ALL and AML pooled, n=76), (C) ALL patients (n=36), and (D) AML patients (n=40).

Supplementary Figure S2

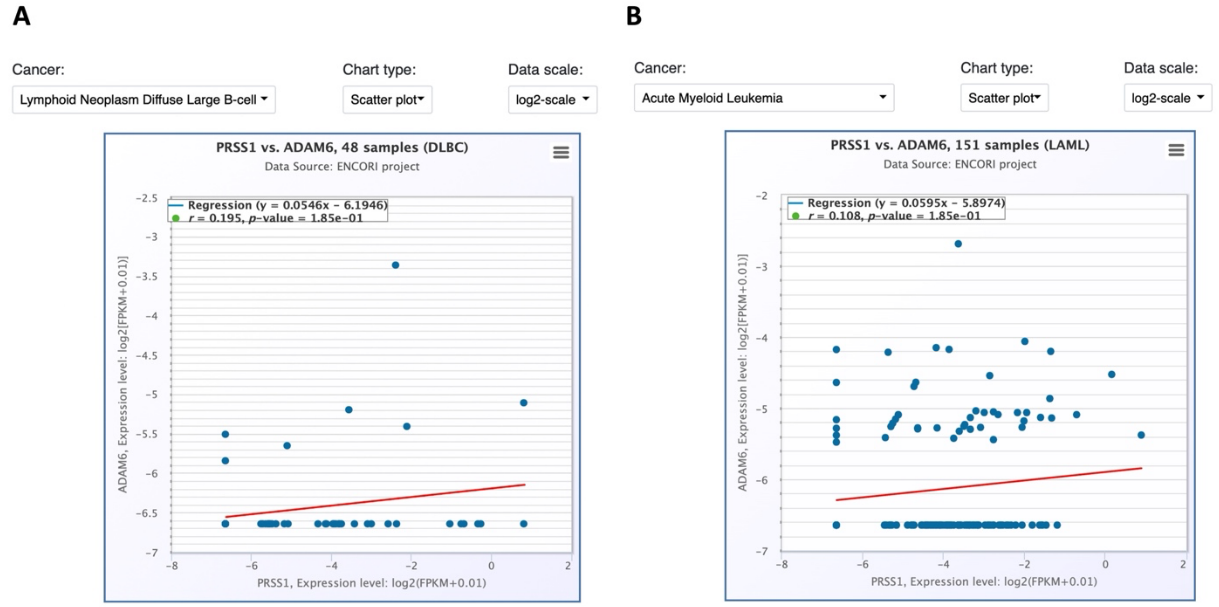

Co-expression of ADAM6 and PRSS1 in (A) Lymphoid neoplasm diffuse large B-cell, and (B) Acute myeloid leukemia datasets on ENCORI Pan-Cancer analysis platform.

Supplementary Figure S3

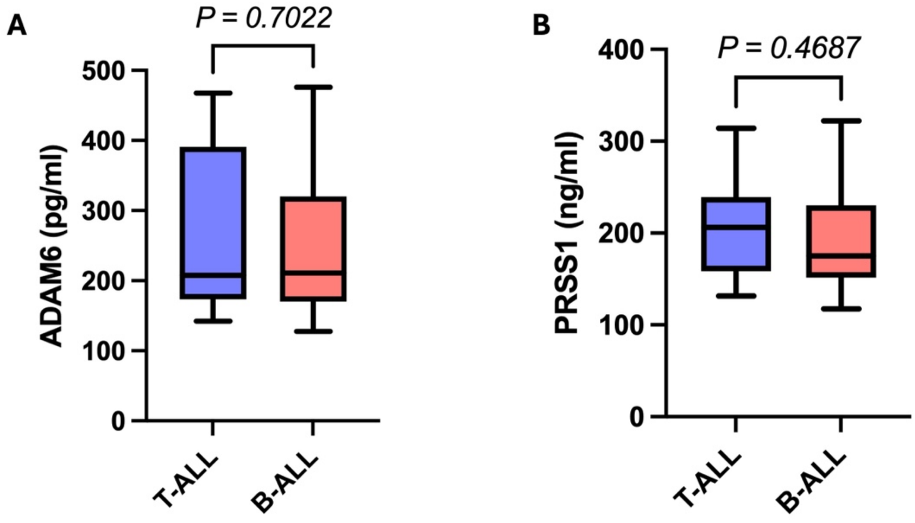

Serum levels of ADAM6 and PRSS1 in T-ALL and B-ALL patients. (A) ADAM6 levels in T-ALL patients (n = 13) compared to B-ALL patients (n = 23). (B) PRSS1 levels in T-ALL patients (n = 13) compared to B-ALL patients (n = 23). Box plots represent the interquartile range, the line inside the box represents the median and the bars represents minimum and maximum values. Shown p-values are for Mann-Whitney test. *ALL, acute lymphoblastic leukemia; ADAM6, A Disintegrin And Metalloproteinase Domain-6; and PRSS1, Serine protease 1.*

Supplementary Figure S4

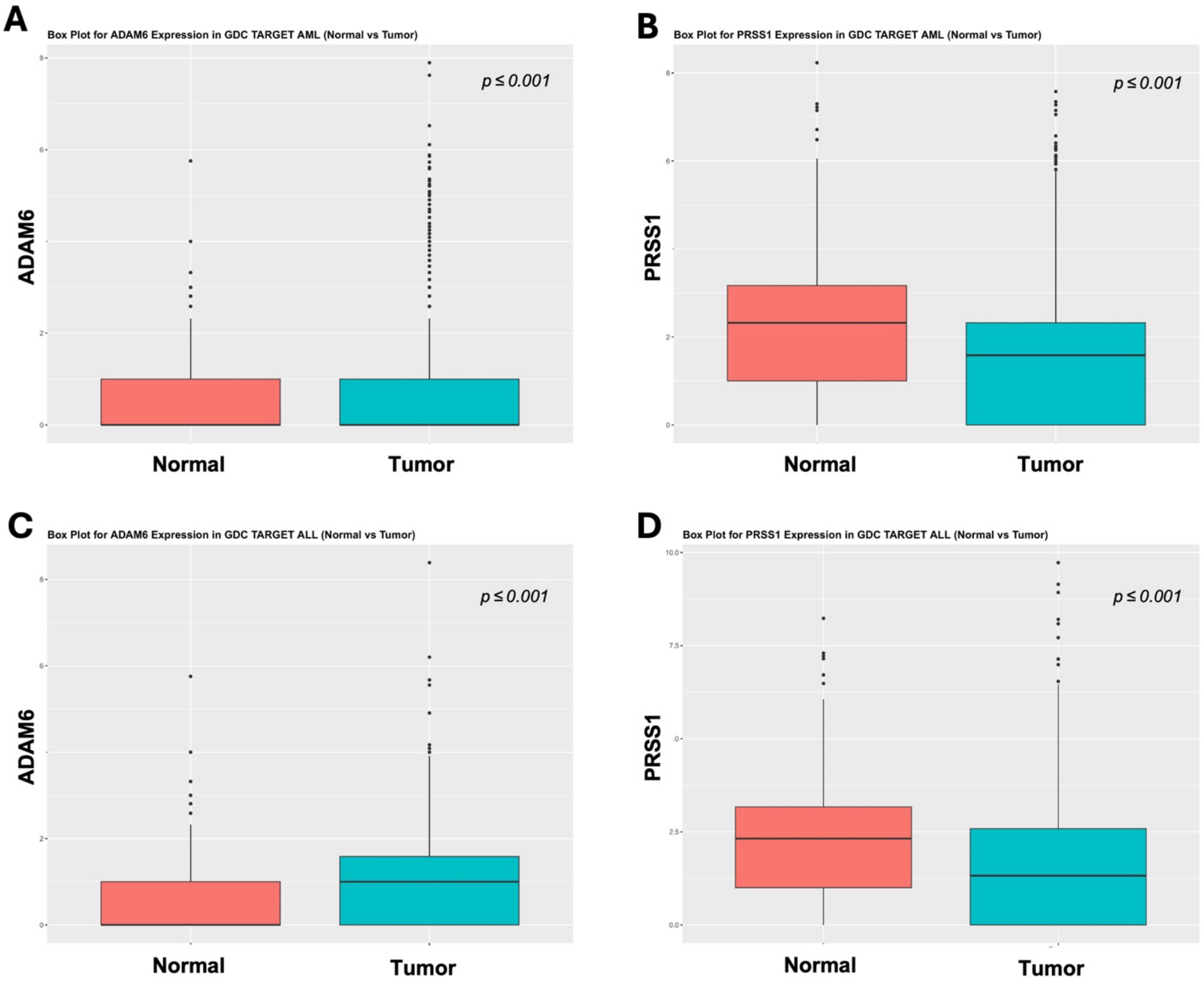

Box plots for ADAM6 and PRSS1 expression in GDC TARGET acute leukemia tumor samples compared to normal samples. (A) Box plot for ADAM6 Expression in GDC TARGET AML, (B) Box plot for PRSS1 Expression in GDC TARGET AML, (C) Box plot for ADAM6 Expression in GDC TARGET ALL, and (D) Box plot for PRSS1 Expression in GDC TARGET ALL. *ALL, acute lymphoblastic leukemia; AML, acute myeloid leukemia; ADAM6, A Disintegrin And Metalloproteinase Domain-6; GDC, Genomic Data Commons; TARGET, Therapeutically Applicable Research to Generate Effective Treatments; and PRSS1, Serine protease 1.*
